# Supplementary material for: The nematode homologue of Mediator complex subunit 28, F28F8.5, is a critical regulator of C. elegans development
Source: PeerJ. 2017 Jun 6;5:e3390. doi: 10.7717/peerj.3390 (PMC5464003; doi:10.7717/peerj.3390)
Supplement: Supplemental Information 1 — Sequences of MED28 homologues shown in Fig. 1 are here available in FastA format for direct submission to Promals for analysis using other parameters. [file peerj-05-3390-s001.doc]

**Supplementary File S1. Sequences of multiple sequence alignment of selected metazoan homologues of MED28 with F28F8.5a.**

Sequences compared in Figure 1:

>C.elegans/81-195

PTYLGDAIDSLMMHWCQLLTNVSVKAPVPPPSTLNHVKEVAEVCSKHFRDASVDVNNEFTRLGVQWEMEQPY

SQYAIEEENLDEAIERQETIIAAAREMLNSRIQ-IYNEAHPNAG

>T.spiralis/3-115

-EYNGDVINNFRQAVKSCLTLLSVPVKSRHI-EADEIKTTAEVATHRLIEAARRSERHFVRLYALFSAYCPE

EVLKEEINEMKQEIERKKNMLLKHEEKMIAWEQ-ILSEAETPLT

>W.bancrofti/51-164

DTPLGQAVTNLHGSWAQLISDLSARTGYLPP-TLEHIKEVAECAVRQLKDSCHDLTREFARVGLEWRLTHPD

EALAEDLADYDQAMLRQESLLERAASIVERRLS-DLGAEKNSQG

>P.pacificus/56-169

RSALGDAVDALLLNWSQIMSNTGTKYPSVPP-SLDHVKEVAAIVVQGFRDACGDLNAEFAKVALEWELENGR

AAEDERVAALKGSISRQVTLLARAQAALDLRTE-DYFCSSLDAA

>A.ceylanicum/50-158

RTPLGDAIDSLLVRWAQLLSNVSSRPPVPAPSTIDHVKEVVEISMYHFRDSCLDVCSEFTKIDLQWQLDHPE

EVYEDEMKGLDDALIRQETLLARAHGILDRRCK-EFFG------

>G.pallida/47-158

DSALGTALKNMVMQWNSLLSQVVTEVDHPQP-ILEHIKETAEFSVKQFRDACLAMNSELTRISMDWQLNFPD

EMVKQEIVEYENSIRRQEDLLTKIRQKLDEEI---SSGENSNFG

>P.davidi/142-252

ATPLMLCVDRLYQSYNNLILDFFPESGIALP-PQSDIKESAEFNVKSFRDACQELTAEFTRSAVEWQLMNPQ

EYYADEIKDLDKAIARQTEIQKRVEGKINSEINKSLNGD-----

>I.scapularis/4-114

---SSQIVDDFENSFQACLAAVTNPDYFYVR-DSEEVKTGVEQTIQRFLDVAKQMECFFLQKRLVLSAQKPE

QIVMEDNTELKNELARKEQLLQKYHEKIHFWQS-LLNDTNNAPG

>D.melanogaster/5-118

ESGGGNLMDEFEEAFQSCLLTLTKQEPNSGT-NKEEIDLEVQKTTNRFIDVARQMEAFFLQKRFLVSTLKPY

MLIKDENQDLSIEIQRKEALLQKHYNRLEEWKA-CLSDIQQGVH

>H.sapiens/38-151

RPSSSTLVDELESSFEACFASLVSQDYVNGT-DQEEIRTGVDQCIQKFLDIARQTECFFLQKRLQLSVQKPE

QVIKEDVSELRNELQRKDALVQKHLTKLRHWQQ-VLEDINVQHK

*Caenorhabditis elegans*, O18692; *Trichinella spiralis*, E5RZQ1; *Wuchereria bancrofti*, EJW84794.1; *Pristionchus pacificus*, translated contig of CN657719.1 FG102945.1 CN657262.1 CN656622.1; *Ancylostoma ceylanicum*, A0A016SKV7; *Globodera pallida*, translated CV578368.1; *Panagrolaimus davidi*, translated JZ658977.1; *Ixodes scapularis*, B7PAW5; *Drosophila melanogaster*, MED28_DROME; *Homo sapiens*, MED28_HUMAN
