# Supplementary figures and images for: The nematode homologue of Mediator complex subunit 28, F28F8.5, is a critical regulator of C. elegans development
Source: PeerJ. 2017 Jun 6;5:e3390. doi: 10.7717/peerj.3390 (PMC5464003; doi:10.7717/peerj.3390)

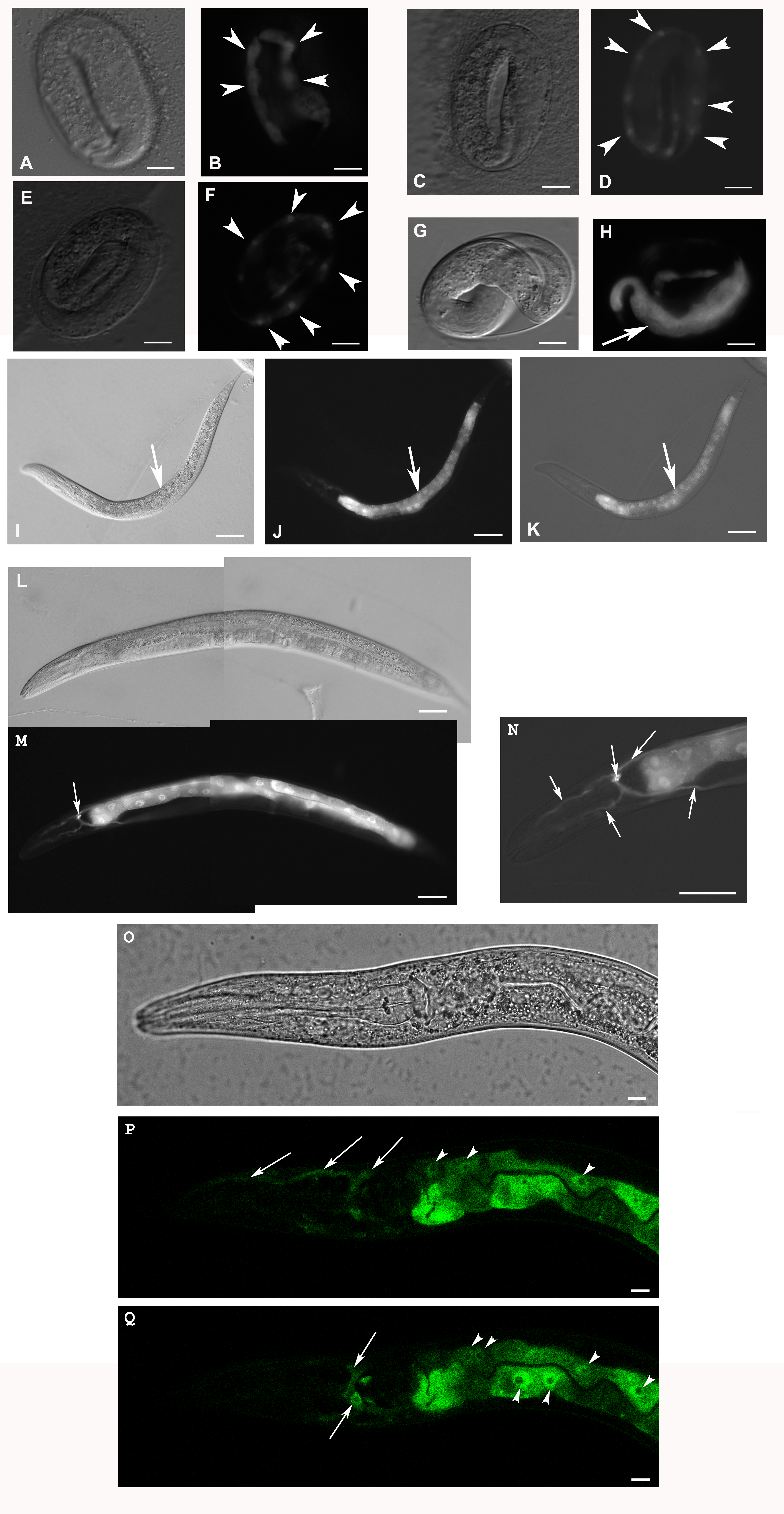

Supplement: Supplemental Information 2 — Panels A, C, E and G show images in Nomarski optics and corresponding panels B, D, F and H in GFP fluorescence. In the twofold stage (panels A and B, C and D), the transgene is expressed in epidermal cells–seam cells (panels B and D). The transgene is localized both in the cytoplasm and nuclei (panel B, arrowheads) or is found accented in nuclei (panel D, arrowheads, contrast and brightness were increased +20, original picture is provided as Raw Data). In the threefold embryo (panels E and F), the transgene is progressively localized in nuclei (arrowheads) as well as in the cytoplasm of epidermal cells. Panels G and H show a threefold embryo before hatching with the expression of the transgene predominantly in the cytoplasm of intestinal cells (arrow). Panels I (Nomarski optics), J (GFP fluorescence) and K (brightfield microscopy together with recorded GFP fluorescence) show a L3 larva in which the nuclear localization of F28F8.5::GFP becomes more accumulated in nuclei of enterocytes (arrows). Panels L, M and N show an adult hermaphrodite animal with F28F8.5::GFP fluorescence in nuclei of enterocytes and in the excretory cell and its channels (arrows). Panels O to Q show the proximal part of the body of a hermaphrodite in L3 stage in confocal microscopy (panels P and Q are parallel optical planes) and an image in Nomarski optics (panel O). Upper arrows indicate the excretory channels and the lower arrow points to the the body of the excretory cell (in panel Q). Arrowheads indicate nuclei of enterocytes with accumulated F28F8.5::GFP surrounding large nucleoli. F28F8.5::GFP is also localized diffusely in the cytoplasm of enterocytes. Bars represent 10 μm. [file peerj-05-3390-s002.png]

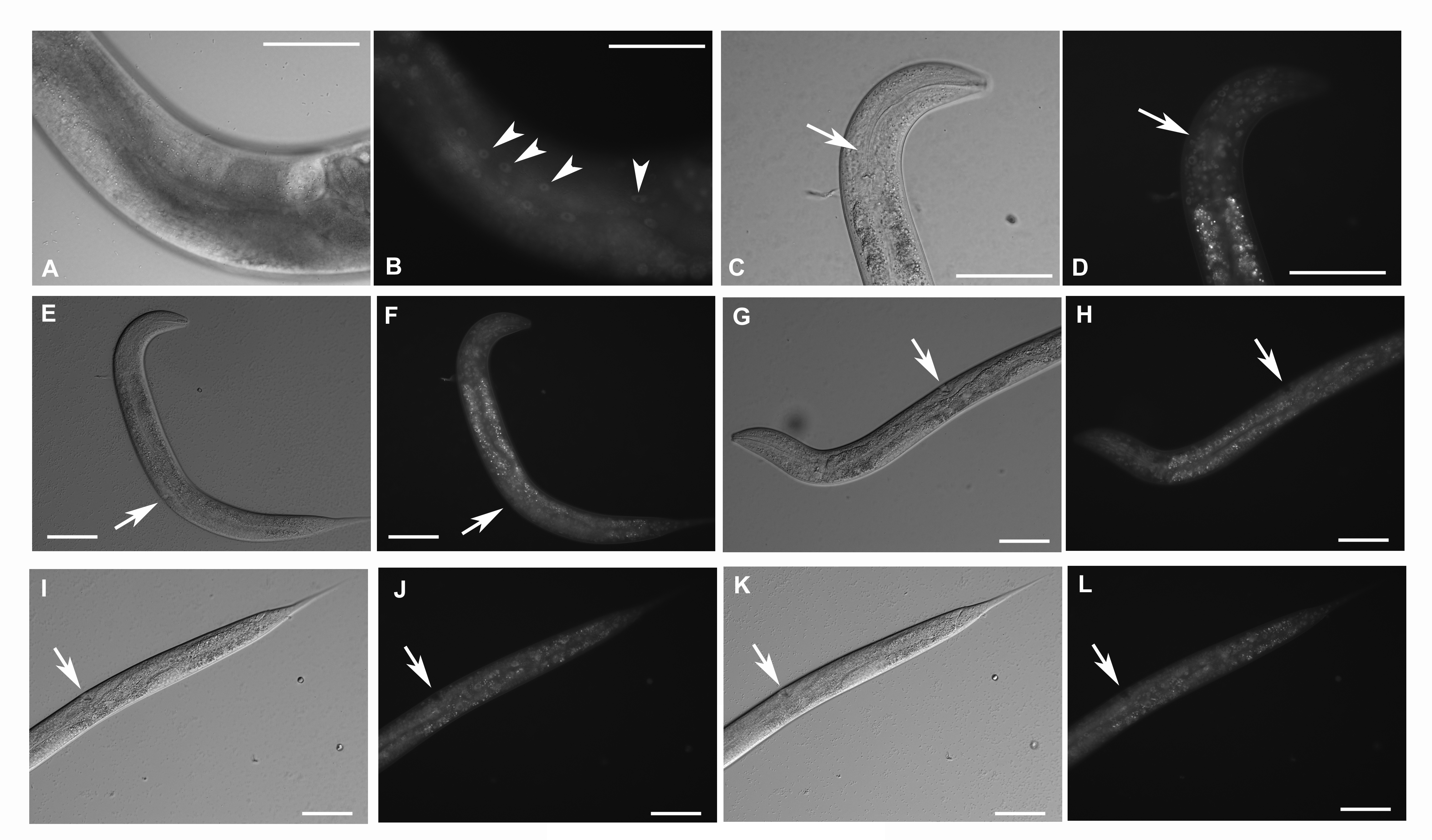

Supplement: Supplemental Information 3 — Panels A and B show part of the body of an adult hermaphrodite in focus on epidermal cells in Nomarski optics (A) and GFP fluorescence (B). Arrowheads mark GFP signal in nuclei of epidermal cells in panel B. Panels C to L show two L3 larvae (one in panels C to F and second in panels G to L). Panels C, E, G, I and K are in Nomarski optics and correspond to panels D, F, H, J and L in GFP fluorescence in the same focal planes. Pharyngeal cells shown in panel D express GFP::F28F8.5 predominantly in nuclei (marked by an arrow). Panels F, H, J and L show cells of the developing vulva expressing GFP::F28F8.5 predominantly in nuclei shown in 3 focal planes (marked by arrows). Bars represent 50 μm. [file peerj-05-3390-s003.png]

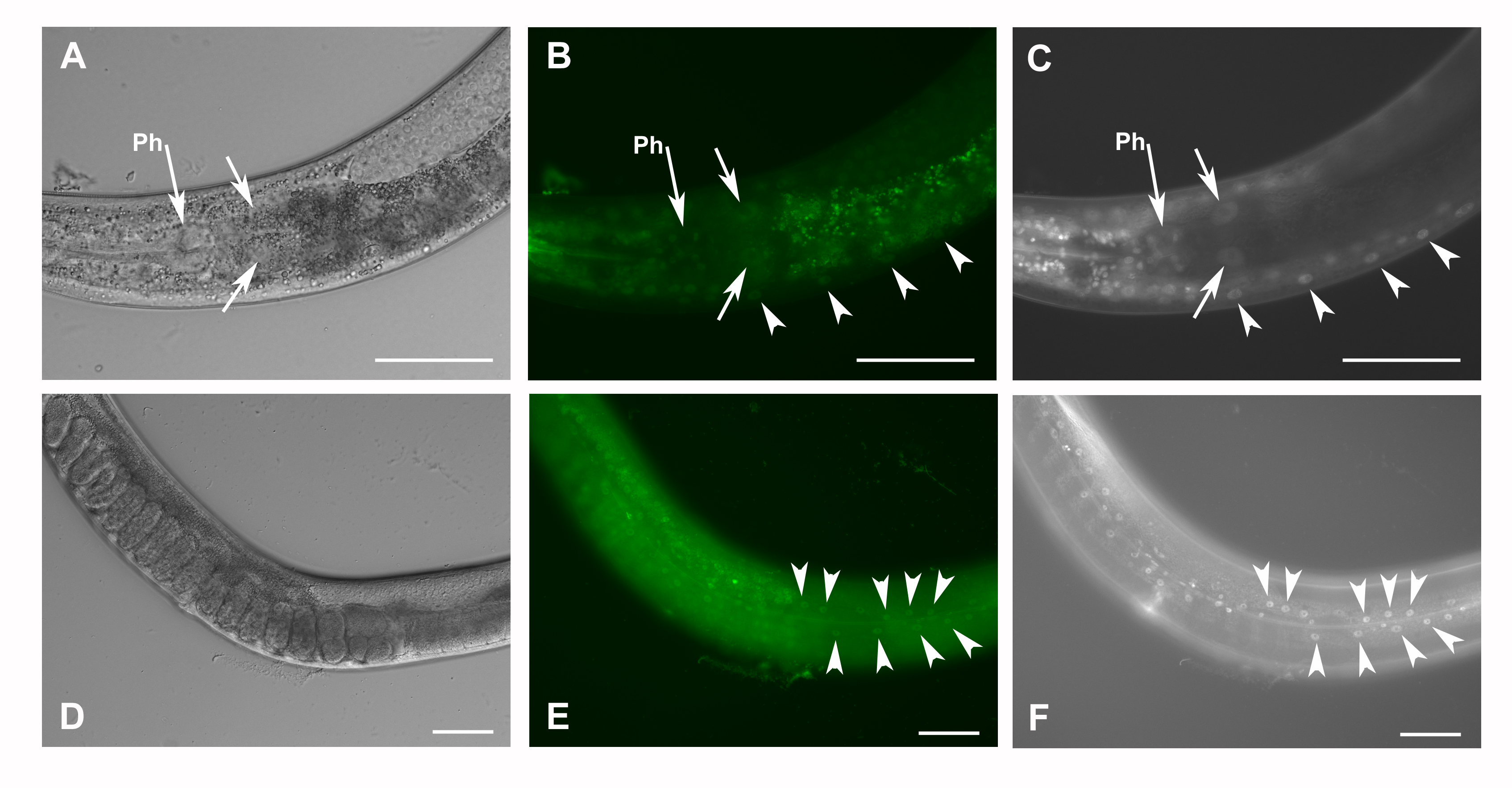

Supplement: Supplemental Information 5 — Homozygous hermaphrodites carrying gfp::F28F8.5 edited gene were observed in Nomarski optics (panels A and D), GFP fluorescence (panels B and E) and DAPI staining (panels C and F). The head area with the pharynx (indicated by long arrows with Ph) is showing nuclei of pharyngeal muscle cells labeled by both GFP fluorescence (B) and DAPI fluorescence (C). Short arrows in panels A, B and C indicate two large nuclei of enterocytes with labeled areas by both GFP fluorescence (B) and DAPI fluorescence (C). Similarly, the neurons of the neuronal cord have nuclei positive in both GFP fluorescence (B) and DAPI fluorescence (C) marked by arrowheads. Panels D, E and F show an adult hermaphrodite in focus on epidermal cells. Arrowheads mark nuclei of epidermal cells positive in both GFP fluorescence (E) and DAPI fluorescence (F). Bars represent 50 μm. [file peerj-05-3390-s005.png]

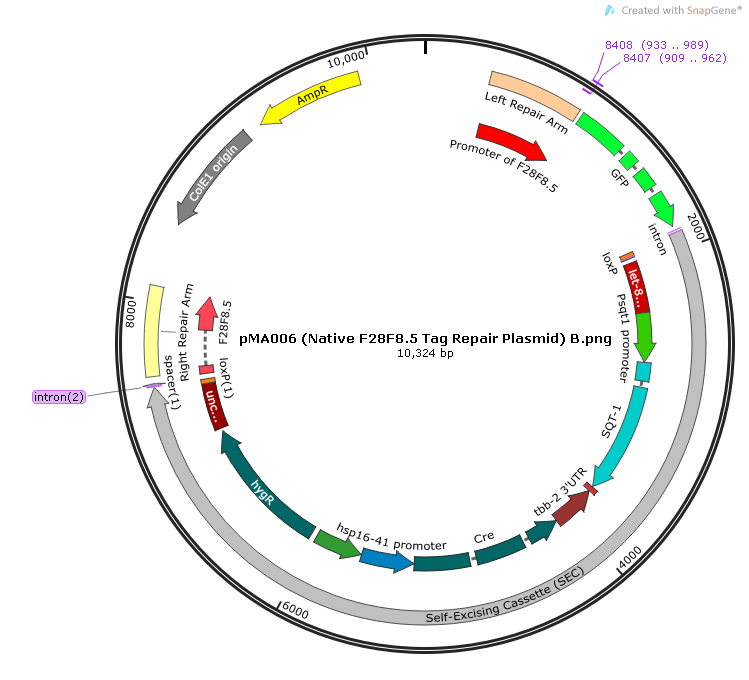

Supplement: Supplemental Information 7 — Scheme of the repair template plasmid pMA006 designed using SnapGene software (from GSL Biotech; available at snapgene.com). [file peerj-05-3390-s007.png]

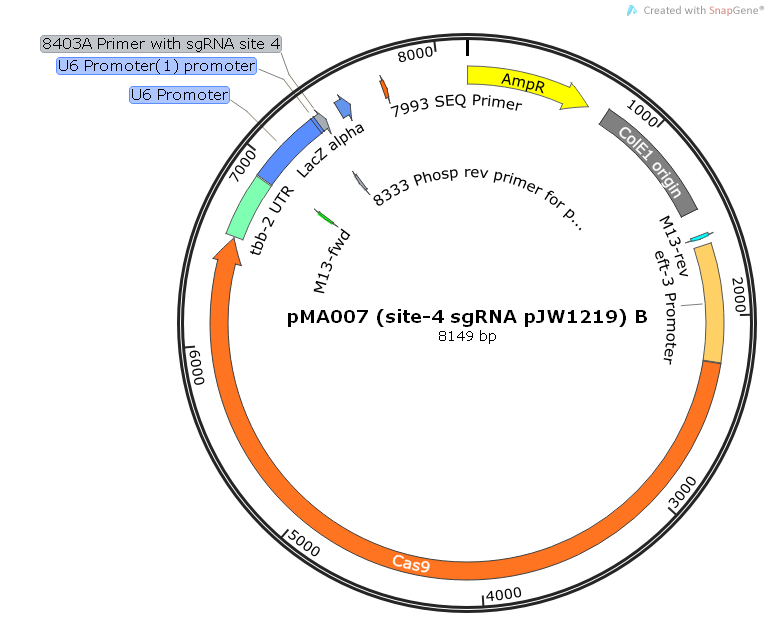

Supplement: Supplemental Information 8 — Scheme of the repair template plasmid pMA007 designed using SnapGene software (from GSL Biotech; available at snapgene.com). [file peerj-05-3390-s008.png]

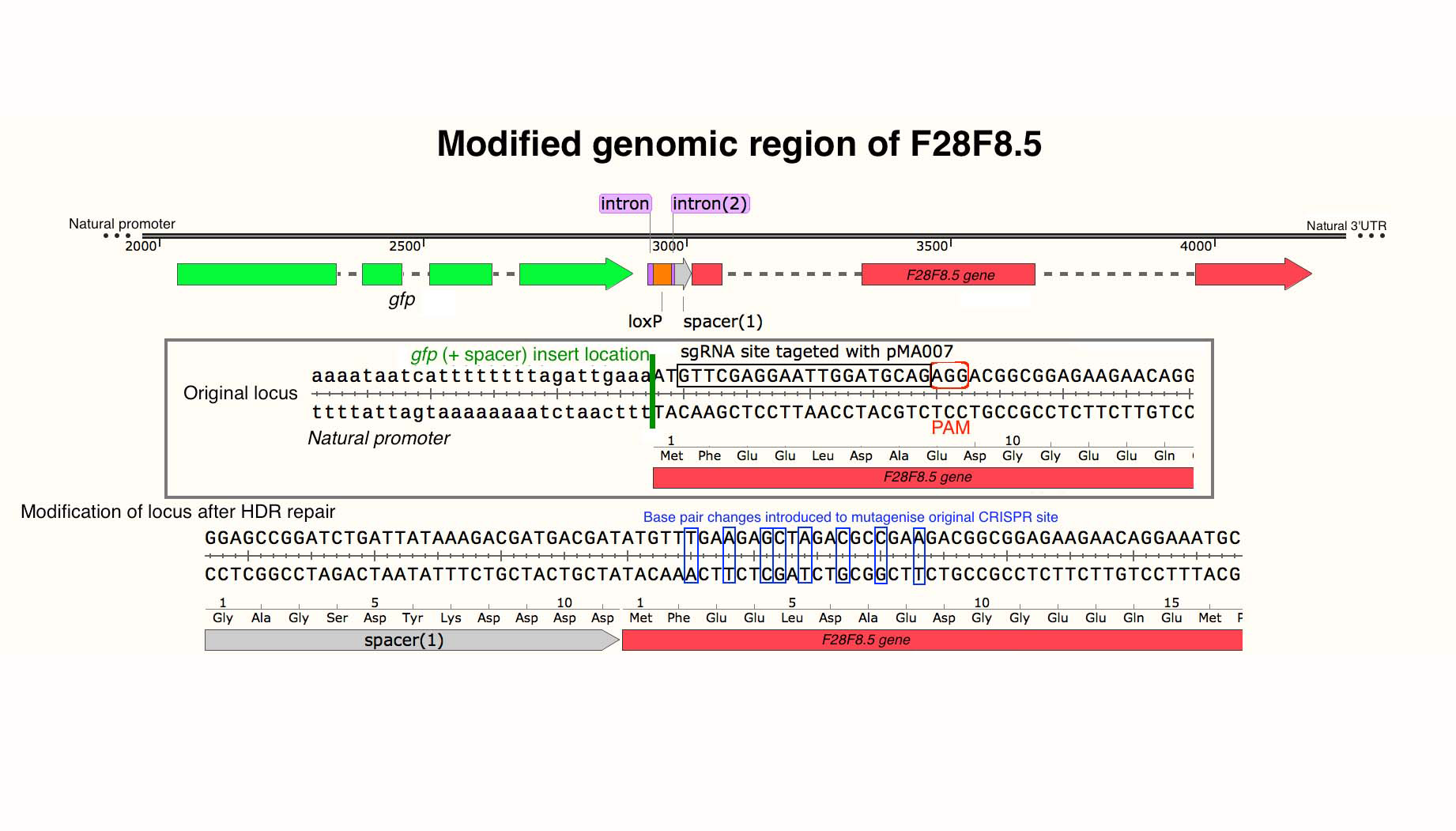

Supplement: Supplemental Information 9 — Scheme of the modified genomic region of F28F8.5 designed using SnapGene software (from GSL Biotech; available at snapgene.com). [file peerj-05-3390-s009.png]

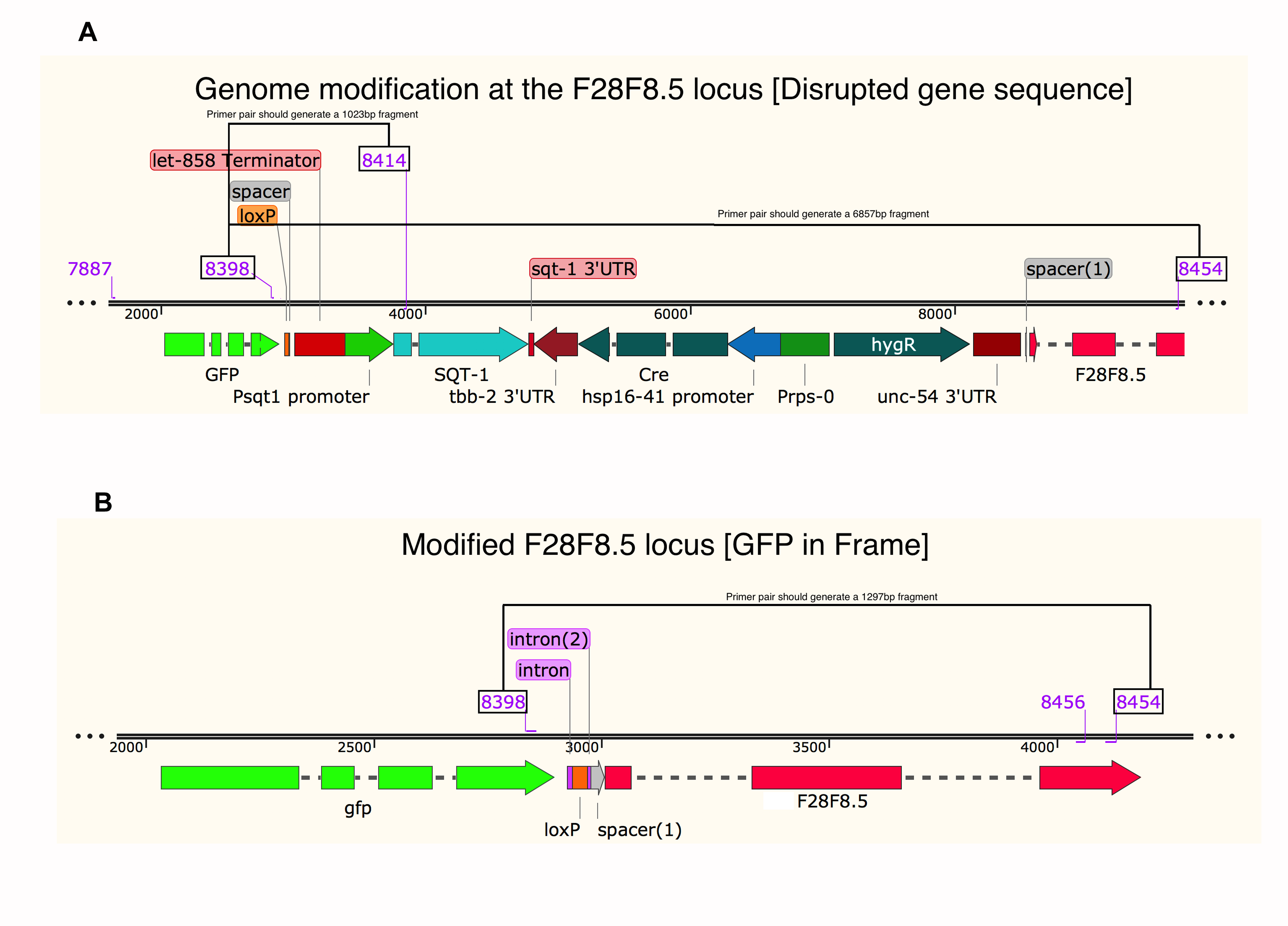

Supplement: Supplemental Information 10 — Scheme of F28F8.5 genome editing designed using SnapGene software (from GSL Biotech; available at snapgene.com). [file peerj-05-3390-s010.png]
