# Supplementary material for: The nematode homologue of Mediator complex subunit 28, F28F8.5, is a critical regulator of C. elegans development
Source: PeerJ. 2017 Jun 6;5:e3390. doi: 10.7717/peerj.3390 (PMC5464003; doi:10.7717/peerj.3390)
Supplement: Supplemental Information 4 [file peerj-05-3390-s004.docx]

**List of primers**

6232 AGCTTGCAGCCTGAGGTCGACT

6233 AAGGGCCCGTACGGCCGACTAGTAGG

6234 GGAAACAGTTATGTTTGGTATATTGGG

7721: AGTCGGTGTGCGAAGGAG

7722: GATAGCGACCGCATCCAC

7889 ATGTTCGAGGAATTGGATGCAG

7890 TTAAGTGGTAAAATGTTTCCCAGC

7886 TTCGACGCCGCCAGGAGCTTCAG

7887 TCAAATGCCGAACAACAGCGCAG

7888 AGTCGACCTGCAGGCATGCAAGCTAGTGGTAAAATGTTTCCCAGC

7964 caattatgcaactatggccttcc

7966 agtcgacctgcaggcatgcaagctcattttcgaatttttgctctgaaatt

8255: tggttccgcgtggatccccgATGTTCGAGGAATTGGATGCAG

8256: agtcagtcacgatgaattccTTAAGTGGTAAAATGTTTCCCAGCA

8277: TAATAGAGTCGACCCGGGCGGCCGC

8278: ACCCATGGTGGCGAGGTACCACGCGTGAATTCTCGAGTG

8292: ATGATGCCACGAATGGGACCTCCAG

8293: TTATTGTTTTGACGTTGTTCTGGCAG

8302: ATGAATCCGAATCAACCCCCAAAT

8333 *P-*CAAGACATCTCGCAATAGGAGG

8334 CGACTGGCCGTCGTTTTACAACG

8335 ATAACATGGTCATAGCTGTTTCCTGTGTG

8398 CATGGTCCTCCTCGAGTTCGTCAC

8403A GTTCGAGGAATTGGATGCAGGTTTAAGAGCTATGCTGGAAACAGC

8403B AATGAGGCACATCCAAATGCGTTTAAGAGCTATGCTGGAAACAGC

8404 GTCACGACGTTGTAAAACGACGGCCAGTCGGGCGATAGCTTTTGCAATGTCTATGGAAAC

8405 TCACACAGGAAACAGCTATGACCATGTTATCGTCAGCAGCTGACACCAATGCATC

8406 ATGTTTGAAGAGCTAGACGCCGAAGACGGCGGAGAAGAACAGGAAATGCCAATG

8407 TTTCAATCTAAAAAAAATGATTATTTTTTCCAAAAACTCTAAAATTGAAGGTAG

8408 GAAAAAATAATCATTTTTTTTAGATTGAAAGGAGCATCGGGAGCCTCAGGAGCATCG

8409 GTCTTCGGCGTCTAGCTCTTCAAACATATCGTCATCGTCTTTATAATCAGATCCGGCTCC

8414 CCTCCGAGTAGATGGTTGACATGG

8454 CCGAGAGTTCAACATTTCTCTGGCGG

8455 GAAAAGAAGAAAGAAGAGAGGAACG

8456 CTCCTCTTCAATCGCATATTGTG

8457 CGCTTAACATCACAACATGTTGCGTACAGG

8481 ggcaaagtcggtagagccactgcc

8502 aaagttattctggcattccatgag

8519: ACGGAGCTCGAATTCGGATCCGCGAC

8520: CGACAAGCTTGCGGCCGCACTC

8527: CTTATCGTCGTCATCCTTGTAATCGACTTGATCATCGGTTTTC

Primers for quantitative PCR

probe 133

7889: ATGTTCGAGGAATTGGATGCAG

8029: TCGCCGAGAATATCGTCAAT

probe 70

8253: CGGGGATCCACGCGGAACCAGATC

8254: GGAATTCATCGTGACTGACTGACGATCTGC

(Note *P*- stands for synthetically added 5’-pentaphosphate)
